# Methods of Thermal Analysis as Fast and Reliable Tools for Identification and Quantification of Active Ingredients in Commercially Available Drug Products

**DOI:** 10.3390/pharmaceutics17091099

**Published:** 2025-08-23

**Authors:** Marek Wesolowski

**Affiliations:** Department of Analytical Chemistry, Faculty of Pharmacy, Medical University of Gdansk, Gen. J. Hallera 107, 80-416 Gdansk, Poland; marwes@gumed.edu.pl; Tel.: +48-58-349-15-25

**Keywords:** DSC, DTA, TGA, active pharmaceutical ingredients, excipients, drug products, non-compliant products, distinguishing, qualitative identification, quantification

## Abstract

**Background/Objectives**: Drug products on the pharmaceutical market must meet a number of requirements that guarantee their quality, safety, and efficacy. Accordingly, periodic inspection of the content of active pharmaceutical ingredients (APIs) in marketed drug products is carried out, confirming that they meet all quality and quantity requirements for a given drug formulation before the expiration date. Therefore, the purpose of this study was to evaluate the suitability of the most commonly used thermal analysis methods, differential thermal analysis (DTA), differential scanning calorimetry (DSC), and thermogravimetric analysis (TGA), in the control of the composition of commercially available drug products. **Results**: Based on a review of the literature, it was shown that thermal methods can be useful in distinguishing drug products from different manufacturers, which guarantees their usefulness in quality control of finished drug products and detecting drug products from illegal manufacturers. They are also useful as tools for confirming the presence of APIs in dosage forms under investigation. The cited literature also indicates that DSC and TGA methods can be used in the quantification of APIs in marketed drug products and to detect non-compliant drug products. The use of chemometric techniques to interpret thermal data can eliminate the adverse effects of excipients on quantification results. **Conclusions**: Thermal methods are a good complement to chromatographic and spectroscopic methods, with the particular advantages of not needing any sample pretreatment, low sample weight, and short analysis time.

## 1. Introduction

One of the most important issues from the point of view of public health is the quality, safety, and efficacy of drugs used to treat, prevent, or diagnose diseases [1]. The importance of this issue is confirmed, among other things, by the fact that the International Council for Harmonization of Technical Requirements for Registration of Pharmaceuticals for Human Use (ICH) was established in 1990 with the intention of standardizing the requirements for drugs placed on the pharmaceutical market [2]. Among other things, the ICH guidelines indicate a number of test and acceptance criteria that should be taken into account by drug manufacturers [3], as their realization guarantees the quality of both active pharmaceutical ingredients (APIs, active substances, drug substances, medicinal substances) [4] and drug products (pharmaceutical preparations, drug formulations, dosage forms) [5]. Active ingredients are responsible for the pharmacological effect, while drug products are complete dosage forms that contain appropriate doses of APIs and are intended for human or veterinary use. According to ICH guidelines, it is important, among other things, to determine the physical and chemical properties of APIs and to identify and quantitatively analyze APIs in drug products [3].

Out of the factors that determine the quality of drugs, the stability of active ingredients [6] and their content in drug products [7] are highly important. Low stability of APIs results in their degradation when exposed to light, oxygen, or moisture, with the formation of impurities [8]. Since impurities can be pharmacologically or chemically active, can enhance or impair the pharmacological efficacy of APIs, and can also sometimes be teratogenic, mutagenic, or carcinogenic, they cause a potential danger to the patient [9]. A consequence of the low stability of APIs can also be a decrease in their content, and thus a decrease in the therapeutic value of drug products. According to relevant regulations, during the permissible shelf-life of drug products, the API content must not decrease to below 95% [7]. Therefore, a periodic check of the content of APIs in commercially available drug products is carried out, the purpose of which is to confirm that the drug products before the expiration date meet all qualitative and quantitative requirements for the drug formulation.

A range of analytical techniques, including chromatographic [10], spectroscopic [11], and electroanalytical [12] techniques, are used in the composition control and quantitative analysis of drug products. The most widely used is high-performance liquid chromatography (HPLC) and many of its types, which allows not only the evaluation of the content of active ingredients, but also enables the identification of APIs, excipients, and unwanted degradation products. In contrast, thermal analysis methods are relatively rarely used. To date, the only article to review the literature related to the use of thermal analysis in the qualitative and quantitative analysis of drug products was published in 1992 [13]. It shows that the most commonly used thermal analysis methods, differential thermal analysis (DTA), differential scanning calorimetry (DSC), and thermogravimetric analysis (TGA), can be useful in the composition control of solid and soft drug products. Curves reflecting the thermal transformation in drug products make it possible to distinguish individual drug products from each other, identify their components, and quantify the content of active ingredients in tablets, capsules, ointments, and creams.

Taking into consideration the above, the purpose of this paper was to evaluate the progress in the use of various methods of thermal analysis (DSC, DTA, TGA) in the control of the composition of commercially available drug products. The significant advances in thermal analysis observed over the past few decades, resulting from, among other things, computerization, miniaturization, automation, and the possibility of coupling several different measuring instruments, have enabled a significant expansion of the research area, contributed to significant improvements in the precision, accuracy, and sensitivity of measurements, and reduced the cost of analysis. This has resulted in the recognition of thermal analysis methods as convenient, rapid, and reliable measurement techniques with wide applications in science and the pharmaceutical industry. Thermal methods are invaluable tools for studying the physicochemical properties of solid-phase APIs, i.e., for studying physical transformations occurring in single-component systems composed of APIs (melting, vaporization, sublimation, crystallization, conversion from amorphous to crystalline state, solid–solid and glass transitions) and chemical transformations (hydration, dehydration, rehydration, desolvation, degradation, decomposition), and to study complex processes occurring in binary or multicomponent systems (eutectics, solid solutions, solid dispersions, co-crystals, inclusion complexes) in which API is one of the components [14,15]. They are recognized methods for testing the purity and polymorphism of APIs, detecting incompatibilities between ingredients (APIs, excipients) at the preformulation stage, and for assessing the kinetics and mechanism of thermal degradation of APIs. However, there is no comprehensive evaluation of the utility of thermal methods in the control of the composition of commercial drug products.

## 2. Methods of Thermal Analysis

According to the recommendation of the International Confederation of Thermal Analysis and Calorimetry (ICTAC) regarding nomenclature in thermal analysis, “Thermal analysis (TA) is the study of the relationship between a sample property and its temperature as the sample is heated or cooled in a controlled manner”, and “a technique exists for each property or physical quantity that is measured versus temperature” [16]. According to these definitions, thermal measurements can include changes in such sample properties as temperature difference, difference in heat flow rate, dimensions and mechanical properties, electrical properties, magnetic properties, acoustic properties, and others. Measuring changes in a selected property leads to a specific thermal analysis technique. Among thermal techniques, differential thermal analysis (DTA), differential scanning calorimetry (DSC), and thermogravimetric analysis (TGA) have gained the most notice. The other techniques are not widely used, as they either require very complex measurement equipment or can only be used to study a specific group of samples. It should be mentioned that modern thermal analysis apparatus provides ample opportunities for the combined use of methods, not only in the area of various thermal techniques, but also with other instrumental techniques [17].

### 2.1. Differential Thermal Analysis (DTA)

**Figure 1:**
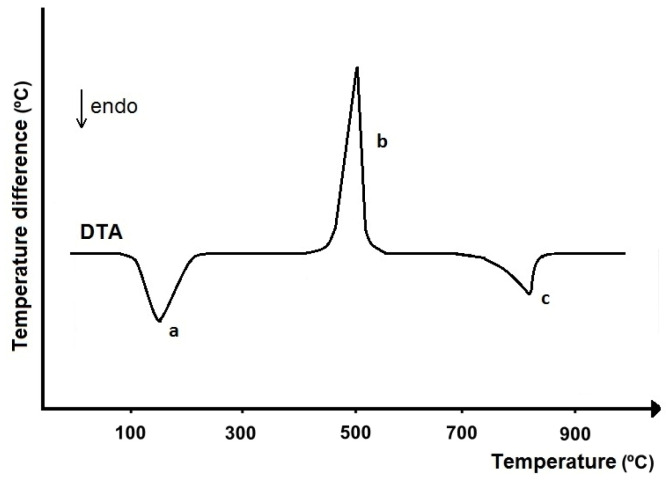
DTA curve of calcium oxalate dihydrate: a—endothermic peak due to dehydration, b—exothermic peak assigned to thermal degradation of calcium oxalate, and c—endothermic peak due to decarboxylation of calcium carbonate.

The principle of DTA is to measure the temperature difference (*∆T*) between a sample (*T_s_*) and a reference substance (*T_i_*) [18]. Both substances are heated simultaneously under the same conditions with a linear increase or decrease in temperature. The resulting temperature difference is recorded as a function of time (*t*) or temperature (*T*), obtaining a DTA curve (Figure 1).

DTA is a dynamic method, that is, one in which a thermodynamic equilibrium state is not reached. In addition, DTA is primarily a method of phase analysis, so the DTA curve reflects the change in the arrangement of phases in the test sample under conditions of controlled increase or decrease in temperature. Using DTA, thermal transformations can be studied that are accompanied by a sufficiently large heat exchange with the surroundings, during which the specific heat changes to a sufficiently large extent in a sufficiently short time. The area of the DTA peak is proportional to the amount of heat exchanged by the sample with the surroundings and depends on the mass of the sample under study, while the shape of the DTA peak is determined by the kinetics of the transformation being studied.

In practice, only those peaks that occur at temperatures lower than the melting point of the test substance are considered characteristic DTA peaks. The presence of a component in a sample can be confirmed by comparing the temperature ranges and the size and shape of the peaks on the DTA curves of the sample under test and the reference substance. This is possible assuming that the measurements were made under the same conditions and with the same type of instrument. Identifying the composition of a mixture is difficult because mutual dilution of the components leads to a reduction in the area of their peaks, often in combination with a change in peak shape. On the other hand, quantitative interpretation of DTA curves requires calibration, i.e., the determination of the relationship between the DTA peak areas the component content in the sample. Calibration is associated with the elimination of the effect of thermal conductivity of the sample and the performance of complex calculations, so classical DTA in this field is increasingly being replaced by DSC.

### 2.2. Differential Scanning Calorimetry (DSC)

**Figure 2:**
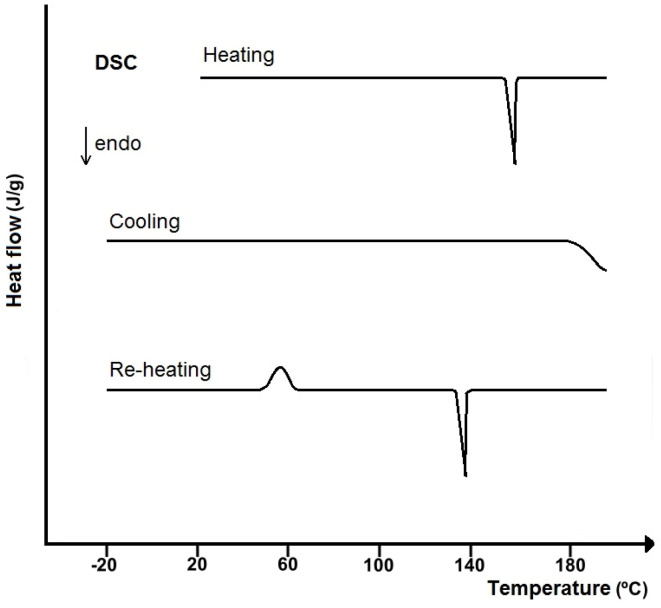
DSC curves of paracetamol polymorphic form I under a heating-cooling-reheating program: heating curve—endothermic peak due to melting; cooling curve—no thermal transitions were seen; and re-heating curve—exothermic and endothermic peaks due to recrystallization and melting of paracetamol polymorphic form II, respectively.

In line with DTA, DSC is a dynamic method in which thermodynamic equilibrium is not reached. DSC is also a method of phase analysis and essentially reflects the changes in the phase arrangement in the sample under study that occur during the measurement. In addition, the DSC curve resembles the DTA curve in its shape, and the enthalpy of the transformation is proportional to the area of the peak under the DSC curve, bounded by the baseline (Figure 2).

DSC is a technique that measures the difference between the rate of heat flow into a sample and into a reference substance, measured as a function of temperature (*T*), while both substances are heated simultaneously under controlled conditions with a linear increase or decrease in temperature [19]. Differences in the rate of heat transfer are caused by physicochemical transformations associated with enthalpy changes (e.g., glass transition, melting, crystallization, degradation). Therefore, DSC is a technique that can be useful for detecting thermal transformations occurring during the heating or cooling of a substance or mixture of substances, and for determining the changes in enthalpy, specific heat, and temperatures at which these transformations occur. The principles for use in composition analysis of single- and multicomponent systems are the same as for DTA.

### 2.3. Thermogravimetric Analysis (TGA)

**Figure 3:**
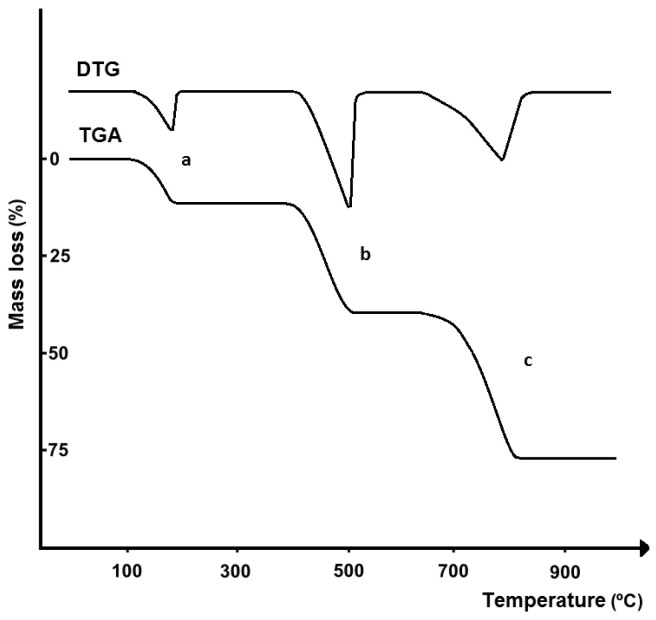
TGA and DTG curves of calcium oxalate dihydrate: a—dehydration with loss of two molecules of crystallization water; b—degradation of calcium oxalate with loss of carbon oxide; and c—decarboxylation of calcium carbonate with loss of carbon dioxide and formation of calcium oxide as a residue.

In thermogravimetric analysis (TGA), the change in sample mass and its loss or gain (*∆m*), occurring during heating or cooling of the sample under controlled conditions, is recorded, and this change is plotted as a function of time (*t*) or temperature (*T*), obtaining the TGA curve [18]. At the same time, the first derivative of the TGA curve can be recorded, obtaining a derivative thermogravimetry (DTG) curve. In this case, the ordinate of the DTG recorder reflects the rate of change of sample mass (*dm/dt*) as a function of time (*t*) or temperature (*T*) (Figure 3). In TGA research, mass loss is most often observed; cases involving mass gain are very rare.

TGA makes it possible to study those physical and chemical transformations that are associated with a change in mass. Identification of a component of a mixture involves comparing the temperature range, curve shape, and mass loss rate of the test substance with the substance used as a standard. This is possible only if the measurements were made under the same conditions and with the same type of instrument. In quantitative analysis, the content of a component in a mixture is estimated from the magnitude of the mass loss.

## 3. Thermal Analysis of Drug Products

Drug analysis is challenging due to the need to detect, identify, and quantify a wide spectrum of active pharmaceutical ingredients and/or their degradation products or other impurities in drug products. There is also a real danger of interference due to the presence in the drug products under study of other substances with similar physicochemical properties to the analyte, and, moreover, that may be present at higher concentration levels than the APIs. For these reasons, the analysis of drug products requires not only a great deal of analytical knowledge, but also a great deal of knowledge in the technology of manufacturing dosage forms.

When planning to use thermal analysis methods in the control of the composition of commercial drug products, it is necessary to take into account the complex composition of dosage forms and the variety of their forms; classic tablets (coated or uncoated), tablets with controlled-release APIs, capsules containing powders or prolonged-release granules, and pellets, dragees, or suppositories. The complex composition of drug products is due to the fact that, in addition to APIs, they contain numerous excipients [20]. The use of excipients is essential, because without their help it would not be possible to prepare suitable dosage forms that will make the APIs contained in drug products resistant to external agents that cause their degradation, resist gastric fluids, do not cause vomiting and nausea, and mitigate the undesirable taste and odor of APIs in the case of orally administered drugs. Appropriate selection of excipients ensures that high concentrations of APIs are achieved at the target site and that dosage forms with delayed or prolonged therapeutic effects can be produced.

As a rule, the composition of drug products includes several to a dozen excipients, for example, most often lactose monohydrate, starch (potato, corn or rice, carboxymethyl starch, or pregelatinized starch), cellulose derivatives (cellulose acetate, carboxymethyl cellulose, ethylcellulose, methylcellulose, hydroxypropyl methylcellulose, or microcrystalline cellulose), gelatin, polyethylene glycols, polyvinylpyrrolidone, talc, and magnesium stearate. It should be remembered that the DTA, DSC, and TGA curves of drug products reflect the physical and chemical transformations undergone by all ingredients contained in the tablets, capsules, ointments, or suppositories under study during heating, including excipients. Therefore, for the correct interpretation of DTA, DSC, and TGA data, information on the type and content of all components contained in the dosage forms under study, i.e., API and excipients, is needed.

**Figure 4:**
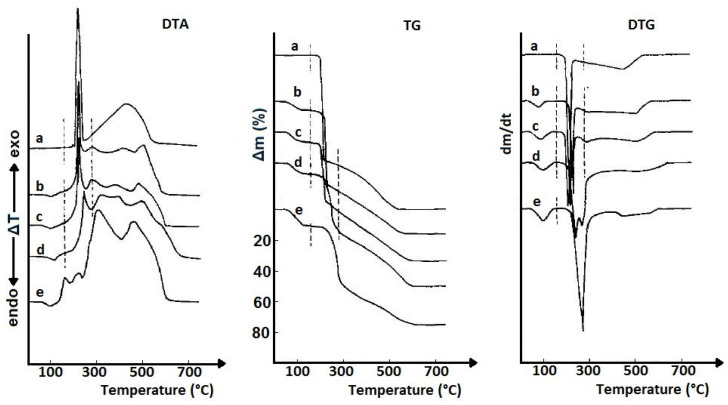
DTA, TGA, and DTG curves of thermal degradation of: a—nitrofurantoin, e—tablet mass containing starch, magnesium stearate, and talc, and its mixtures containing: b—45%, c—30%, and d—15% of nitrofurantoin. The temperature range at which thermal degradation of nitrofurantoin occurs is indicated by the dashed line. Reprinted with permission from Ref. [21], 2025, Springer.

The impact of excipients on the thermal degradation of nitrofurantoin (an antibacterial drug used to treat urinary tract infections) is illustrated by DTA, TGA, and DTG research of mixtures containing starch, magnesium stearate, and talc as excipients [21]. As shown in Figure 4, the DTA, TGA, and DTG curves of the analyzed samples primarily reflect the thermal effects related to the degradation of the ingredient present in the highest amount, i.e., nitrofurantoin, whose content in the analyzed mixtures varied from 15 to 45%. As the amount of API in the mixtures decreased and the content of excipients increased, the DTA peaks and mass loss in the TGA curves associated with the release of volatile degradation products became less and less pronounced. Finally, with several percent nitrofurantoin in the mixture, it is difficult to clearly indicate thermal effects reflecting its presence on the DTA, TGA, and DTG curves.

The relationship between TGA results and the presence of excipients in drug products was also pointed out by Gucluyildiz et al. [22]. Using isothermal TGA, the impact of selected excipients, i.e., lactose, starch, hydroxypropyl methylcellulose, ethylcellulose, polyvinylpyrrolidone, and gelatin, on the release of nitroglycerin (a drug used in ischemic heart disease) from sublingual tablets was studied. The results of the study, confirmed by chemical analysis, showed that the volatility of nitroglycerin depends on the type of excipients and the ratio of concentrations in which they occur in drug formulations.

## 4. Qualitative Analysis of Drug Products

In accordance with the regulations included in the pharmaceutical law, all finished drug products are subject to numerous tests, including qualitative and quantitative composition control, before being released into the pharmaceutical market. Various analytical techniques are used to carry out these tests. Thermal analysis methods can also be helpful, and research into the use of these methods in the analysis of commercially available drug products has a long history, dating back to the 1970s.

### 4.1. Distinguishing Between Drug Products

The first articles reporting DTA, DSC, and TGA results of commercially available drug products (tablets, capsules, and powders) were published by Wendlandt and Collins [23,24,25]. An analysis of 37 solid drug products used as analgesics, gastric juice antacids, and vitamins, as well as selected mixtures of APIs and excipients, showed that similar tablets manufactured by different manufacturers could be distinguished from DTA, DSC, and TGA curves, providing thermal analysis methods with potential utility in forensics. It was found that due to the lack of accurate information about the composition of the products examined and the complexity of their composition resulting from the presence of different excipients, it is not possible to explain the origin of each peak in the DTA and DSC curves. On the other hand, in the case of a small number of excipients in a drug product, its DTA, DSC, and TGA curves are practically identical to those of the active ingredient present in the drug product in question. Comparing the results obtained by these methods, it was further found that more useful information is provided by DTA and DSC curves than by TGA.

The potential usefulness of thermal analysis methods in distinguishing drug products from different manufacturers was also pointed out by Brecht et al. [26]. They used high-tech equipment, i.e., a thermogravimetry coupled with an atmospheric pressure photoionization mass spectrometry (TG-APPI-MS), to test 10 tablets of acetylsalicylic acid (an analgesic, antipyretic, and anti-inflammatory drug). The coupling of the TG analyzer with an MS device proved to be extremely advantageous, as it allows not only to study the behavior of APIs or drug products under controlled heating conditions (TGA), but also to identify and determine the chemical structure of volatile degradation products (MS). The two devices are connected by a heated coupler, through which the volatile degradation products are delivered to the mass spectrometer. The MS spectra obtained made it possible to detect acetylsalicylic acid in all tablets examined and to identify tablet manufacturers, since tablets from a given manufacturer are characterized by a specific composition of excipients. Data obtained with TG-APPI-MS indicated that the newly developed analytical technology could be useful both for quality control of finished drug products and for detecting drug products from illegal manufacturers.

**Figure 5:**
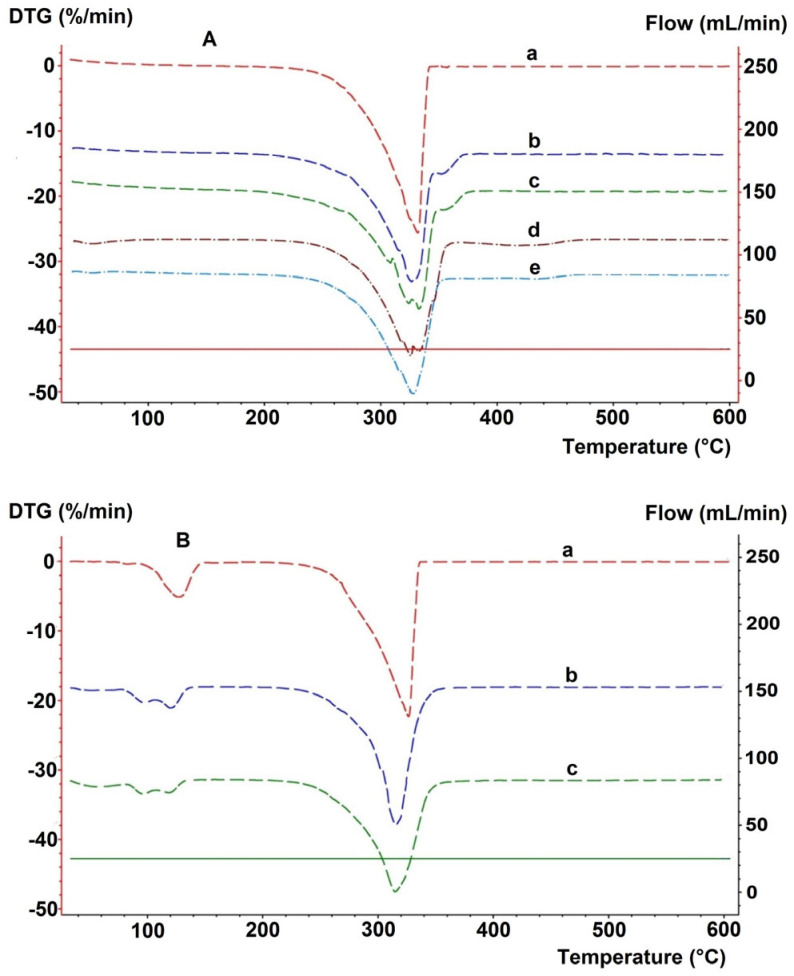
Thermal degradation of active ingredients and tablets containing theophylline and aminophylline. (**A**) DTG curves of: a—theophylline, b—Euphyllin long 300 mg, c—Euphyllin long 200 mg, d—Theospirex retard 300 mg, and e—Theospirex retard 150 mg. (**B**) DTG curves of: a—aminophylline, b—Aminophylline 200 mg, c—Aminophylline 100 mg. Reprinted from Ref. [27].

Studies indicating the possibility of using TGA and DTA to control the composition and clearly distinguish commercial tablets containing theophylline from tablets with aminophylline (a combination of theophylline and ethylenediamine) were conducted by Ramos [27]. Both active ingredients are bronchodilating agents. TGA, DTG, and DTA curves showed that theophylline and aminophylline differ in their thermal degradation profiles; theophylline degrades in one step, while aminophylline degrades in two steps. As shown in Figure 5, this is reflected in the shape of the thermal degradation curves of the commercial tablets, four with different doses of theophylline and two with aminophylline.

Identification of both methylxanthines in drug products would not be possible using the pharmacopoeial method, i.e., UV-Vis spectrophotometry. In this case, both tablets containing theophylline and aminophylline would have absorption maxima at the same wavelength, and their spectra would overlap. This confirms the great advantage of thermal methods over pharmacopoeial methods, in that thermal methods do not require the sample to be dissolved in solution, making it possible to obtain information on the forms in which APIs are present in the drug products under study, i.e., in amorphous or crystalline form, polymorphic form, hydrate or solvate. In addition, the identification of certain excipients provides a potential opportunity to use TGA and DTA to detect non-compliant drug products.

Ramos et al. [28] also applied the above methodology to distinguish between naproxen and its sodium salt (analgesic, anti-inflammatory, and antipyretic agents) in commercial drug products. Taking advantage of the fact that the degradation of the acidic form of naproxen occurs in two steps and its sodium salt in several steps, it is possible to detect which chemical form was used to produce the solid dosage forms and to distinguish tablets containing naproxen acid (four products with different dosages) from tablets containing naproxen sodium (five products with different dosages of naproxen sodium). As in the previous case [27], identification of the acidic form and sodium salt of naproxen in marketed drug products would not have been possible using pharmacopoeial methods such as UV-Vis spectrophotometry and HPLC.

The conditions under which the studies on the use of thermal methods for distinguishing commercial drug products were conducted are summarized in Table 1. They indicate the usefulness of thermal analysis for quickly checking the consistency of drug product composition with that declared by the manufacturer, and, in addition, allow rapid identification of the manufacturer of a given drug product. An interesting example is also the use of DSC in the control of different batches of the multi-ingredient plant preparation from traditional Mongolian medicine (Huricha Liuwei pills), produced by various manufacturers, using multidimensional DSC fingerprinting [29].

### 4.2. Identification of Drug Product Ingredients

A key element of qualitative analysis of finished drug products is confirmation of the presence of active ingredients in the solid dosage forms examined. In an effort to assess the utility of DTA, TGA, and DTG in this regard, 117 commercially available drug products (tablets, granules, capsules, powders, and dragees), used as neuroleptics, expectorants, chemotherapeutics, and vitamins, as well as those containing inorganic active ingredients, were analyzed [13]. The study also included 85 pharmaceutical ointments, which are classified as soft drug forms, and 27 suppositories, which are solid drug forms. Ointments and suppositories differ fundamentally from the other solid dosage forms by the type of excipients, which are, among others, hardened fat, cocoa butter, petroleum jelly, paraffin, lanolin, and many other substances with similar properties [20]. Therefore, their thermal degradation curves, especially the TGA and DTG curves, are similar to each other and differ significantly from the degradation curves of tablets or capsules.

Studies have shown that under certain conditions, DTA, TGA, and DTG curves of thermal degradation can be used to identify APIs in study drug products [13]. In general, identification is made by comparing the shape of peaks and mass losses and the temperature ranges at which they occur on the DTA, TGA, and DTG curves of the sample under study with the same parameters on the curves of the standard substance. The basic prerequisite for a correct comparison of the curves of the sample and the standard is to perform the evaluation using the same type of instrument and the same experimental conditions. Of key importance is the presence of excipients. The temperatures of their phase transformations and thermal degradation should not coincide with the temperatures of the DTA peaks and mass losses (TGA and DTG) of the identified API. The API content of drug products is also important—the higher it is, the more reliable the ability to identify APIs in drug products.

For DTA curves, endothermic peaks due to first-order phase transformations, especially melting, polymorphic changes, sublimation, or evaporation, can be used to identify active ingredients. These are characteristic peaks, most often narrow and high, sharply pointed, run over a narrow temperature range, and have a relatively large surface area that varies in proportion to the API content of the drug product. In contrast, the exothermic DTA peaks associated with the combustion of organic components contained in drug products are of little use. They differ in shape, surface area, and temperature range from the peaks associated with the combustion of individual APIs and excipients. Therefore, they are of little use when identifying APIs.

Compared to DTA, TGA, and DTG curves have less significance in identifying APIs present in drug products, as confirmed by the results of previous studies [23,24,25]. This is due to the fact that the TGA curve reports the quantity of mass loss related to the thermal degradation of the sample under study and the release of volatile degradation products, as well as the temperature range over which this process occurs, but does not indicate what specific chemical degradation reactions of the sample have occurred [13]. For this reason, it is difficult to link a specific mass loss to the degradation of a specific component in the sample under study. This does not include such chemical processes as dehydration of hydrates or decarboxylation of carbonates (see example in Figure 1 and Figure 3). Dehydration and decarboxylation are accompanied by a well-defined mass loss over a known temperature range, clearly visible on TGA and DTG curves. The advantage of TGA is that the DTG curve can be recorded at the same time, which allows more precise observation of any change in the mass loss rate of the sample, facilitating clear separation of successive stages of thermal degradation of drug products.

Pyramides et al. [30] investigated the utility of DSC and TGA to identify atenolol (a beta blocker used to treat high blood pressure and heart-associated chest pain) and excipients in finished tablets. The goal of the combined use of two different thermal analysis techniques was to maximize the ability to detect differences in the composition of the dosage forms studied. The authors assumed that the thermal profile of the examined tablets would be roughly equal to the sum of the tablet component profiles, provided that no interactions between atenolol and excipients occurred. The results showed that a large and narrow endothermic DSC peak confirms the presence of atenolol in the tablets examined. Weak thermal effects associated with the presence of microcrystalline cellulose and sodium starch glycolate are also evident, while any effects reflecting the presence of povidone and magnesium stearate in tablets are absent. It was also shown that the enrichment of finished tablets in magnesium stearate or sodium starch glycolate in amounts ranging from 30 to 50% of tablet mass is reflected by DSC, TGA, and DTG curves, providing a basis for using thermal analysis in checking the consistency of the composition of finished dosage forms with manufacturers’ declarations.

Research on the use of DSC and TGA to identify APIs in commercial drug products was also conducted by Giron and Goldbronn [31]. DSC analysis of 23 drug products (tablets, pellets, capsules, and suppositories) containing various active ingredients showed that APIs can be qualitatively identified in most cases, with this being particularly easy when APIs are present in large doses. Identification involves comparing the onset temperature (*T_on_*) of the endothermic peak of the drug product with the corresponding peak of the API used as a reference substance, analyzed under the same experimental conditions or using literature data. An example is marketed products with paracetamol (an antipyretic and analgesic drug), in which paracetamol can be detected at the 0.08 mg level. However, identification of the API is not possible when its melting peak is covered by the melting peak of lactose monohydrate or when there is an interaction between the ingredients, e.g., the suppository Actifed 300 mg, in which paracetamol interacts with the other APIs, that is, tripolidine hydrochloride and pseudo-ephedrine hydrochloride. Identification is also not possible in the case of drug products containing APIs in low doses and forming a eutectic with excipients present in large quantities (Optalidon and Tonopan tablets). There is then no endothermic DSC peak identifying the API.

In addition, some excipients in drug products, such as lactose monohydrate, β-lactose anhydrous, mannitol, polyethylene glycol, poloxamer, or glycerol monostearate, can be detected using DSC. The possibility of using glass temperature to identify components of the tablet coating was also indicated.

DSC was also used to identify APIs in 27 commercial drug products containing methylxanthines, i.e., theophylline, diprophylline, and caffeine, and other active ingredients [32]. High and narrow endothermic DSC peaks due to melting APIs were used to identify the ingredients of tablets, pellets, granules, capsules, and dragees. The results showed that the identification of APIs in dosage forms is significantly determined by their content. As an example, in tablets containing theophylline amounts of 75 to 95%, the endothermic peak on the DSC curve of tablets is almost the same as the melting peak of theophylline used as a standard. The type and content of excipients also have a significant influence. Of these, lactose monohydrate makes it most difficult to identify APIs. The two endothermic peaks associated with the dehydration and subsequent melting of lactose can overlap with the peaks due to the melting of APIs, especially those found in smaller amounts. As an example, in Euphyllin CR tablets, despite containing 43% theophylline, there is no API melting peak on the DSC curves.

**Figure 6:**
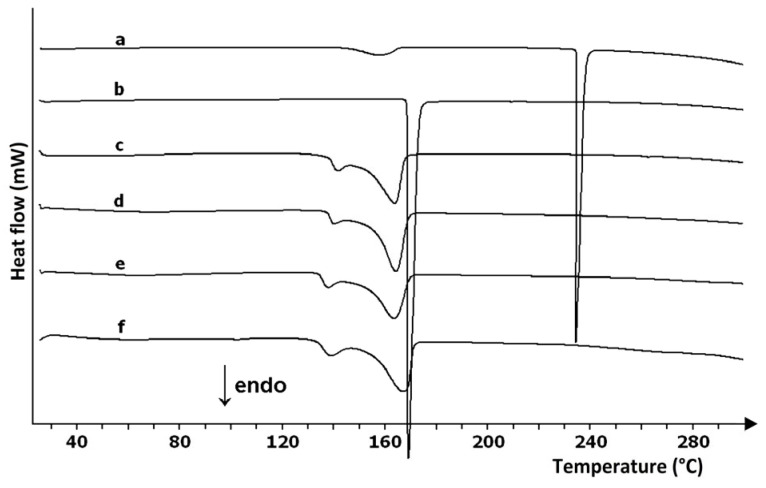
DSC curves of commercial drug products containing caffeine and paracetamol: a—caffeine, b—paracetamol, c—Apap Extra tablets, d—Kofepar tablets, e—Panadol Extra tablets, f—Solpadeine capsules. Reprinted with permission from Ref. [32], 2025, Springer.

Unlike theophylline and diprophylline, the presence of caffeine was not confirmed in any of the 16 drug products examined. The reason could be that the caffeine content of the tablets is too low (3–10%), or it could be because of the presence of other ingredients, APIs, and excipients. Other APIs besides caffeine include, for example, paracetamol (36–80%), acetylsalicylic acid (51–82%), ascorbic acid (4–38%), propylphenazone (22–23%), and ethenzamide (17–19%). Thermal effects from these APIs can overlap with the melting peak of caffeine or can form a eutectic with caffeine, such as paracetamol [33,34]. The effect of the eutectic formed between paracetamol and caffeine on the shape of the DSC curves of drug products is illustrated in Figure 6. The fact of eutectic formation is confirmed by the DSC curves of all drug products containing caffeine and paracetamol. Eutectics can also form between the other ingredients of drug products, such as paracetamol and propylphenazone [34]. Also, DSC curves of Cefalgin and Saridon tablets containing the same quantities of caffeine, paracetamol, and propylphenazone indicate the formation of eutectics. Despite the same content of APIs, the DSC curves of Cefalgin and Saridon tablets differ because they are produced by different manufacturers using different excipients.

At the same time as the DSC measurements, infrared spectra, i.e., Fourier transform infrared spectroscopy (FTIR) and Raman spectra, were recorded for all drug products, and APIs and excipients were examined [32]. To identify the APIs in the drug products, the so-called matching coefficient was used, which, expressed as a percentage, determines the degree to which the spectrum of a given drug product matches the spectrum of the methylxanthine it contains. Comparing the three techniques used, it was found that the endothermic DSC peaks associated with phase transformations were the most useful for identifying APIs in dosage forms. In contrast, the results obtained with Raman spectroscopy were the least useful. The matching coefficients were twice as low as those obtained from FTIR spectra, and in the case of caffeine products, several times lower. The only exception was drug products containing acetylsalicylic acid, for which the matching coefficients were several times higher than those generated from FTIR spectra.

The aim of the following study was to see to what extent DSC, FTIR, and Raman spectroscopy could be used to detect both organic and inorganic magnesium compounds in 14 commercial drug products and 16 dietary supplements [35]. The results showed that in most cases, magnesium compounds can be detected in the evaluated products. For DSC, endothermic peaks associated with dehydration are useful. An example is the DSC curve of Dipromal 200 mg (antiepileptic drug product), which, when compared with the curve of magnesium valproate used as a standard, confirms the presence of magnesium salts in the sample. Similarly, the DSC curve of Bio-Magnesium (dietary supplement) shows an endothermic peak associated with the dehydration of magnesium acetate. Some substances, such as magnesium carbonate and magnesium hydroxide, do not undergo any thermal transformations in the temperature range studied, making them impossible to detect. In the case of FTIR and Raman spectra, the matching coefficient works well as a way to confirm the presence of magnesium compounds in the products. In addition, complex composition and low content were found to be the primary factors hindering the detection of magnesium compounds in products under study.

Miltyk et al. [36] pointed out the potential usefulness of chemometric techniques for constructing discriminatory models to determine the presence of a particular API, or lack thereof, in marketed drug products. The study used DSC curves of 11 active ingredients and 21 over-the-counter tablets purchased from a local drugstore. The DSC curves were preprocessed by standard normal variance (SNV) treatment, and the resulting data were used to construct a matrix containing *n* rows (DSC signals) and *p* columns (DSC curves). The result of processing the DSC data matrix using partial least squares discriminant analysis (PLS-DA) was the development of a model to detect acetaminophen (acetaminophen is a USAN name, used in the US pharmacopoeia, and paracetamol is an INN name, used in the European pharmacopoeia) in the examined tablets. Five PLS factors were considered due to the appropriate proportion between “positive” and “negative” training samples. The resulting discriminant model was positively validated using an external prediction set. However, the results indicate that the application of chemometric techniques for processing DSC data requires further research. More information about the principles of chemometric techniques can be found in the monograph written by Otto [37].

Table 2 summarizes the experimental conditions under which studies were conducted on the use of thermal methods for qualitative identification of APIs in commercially available drug products. As an aside to this work, mention should be made of the study by Cook and Hildebrand [38], who studied the thermal decomposition of 12 sulfonamides (antibacterial drugs) in the temperature range of 25–800 °C. They showed that different substituents at the nitrogen atom of the sulfonamide group generate a unique shape of TGA curves for each compound. Accordingly, they proposed TGA as a rapid method for qualitative identification of sulfonamides by comparing the TGA curves of unknown sulfonamides with those of their standards recorded under the same conditions. They also suggested that further research should move toward the use of TGA for qualitative identification of APIs in mixtures and possibly for quantification of the APIs.

## 5. Quantification of APIs in Drug Products

Quantifying the content of active ingredients in drug products is one of the most important tests before releasing dosage forms into the pharmaceutical market. For this purpose, drug manufacturers use the applicable national pharmacopoeial methods or manufacturers’ methods [7]. Dynamic advances in instrumental analysis lead to the development of further analytical techniques, which are characterized by increasingly better selectivity, precision, accuracy, and sensitivity. These then find application in the quantitative analysis of APIs. Such an opportunity also arises for thermal analysis methods, i.e., DSC and TGA, which can provide a good complement to chromatographic and spectroscopic methods. A particular advantage of thermal methods is the lack of need for any sample pretreatment, low sample mass, and short analysis time.

### 5.1. DSC Measurements

Literature data indicate that the enthalpy of the phase transformation reflected by the DSC endothermic peak area (DTA) is proportional to the mass of the substance undergoing the transformation [19]. The above fact was used by Khattab et al. [39] to determine the content of six sulfonamides in mixtures with alumina (Al_2_O_3_). It was shown that the area of the endothermic DTA peak due to the melting of a sulfonamide, determined with a planimeter, is directly proportional to its content in the mixture and passes through the origin of the coordinate system. On this basis, the content of sulfacetamide sodium, sulfaguanidine, sulfamethoxypyridazine, sulfathiazole, sulfisomidine, and sulfizoxazole was determined in quantities ranging from 30 to 100 mg with an accuracy within 99–101%. In addition, the exothermic DTA peak (T_p_ = 220 °C) associated with the thermal degradation of sulfizoxazole was also used for quantitative analysis. Based on this peak, sulfizoxazole was determined in a quantity of 6–30 mg with an accuracy of ~99%.

Becket et al. [40] employed the DSC procedure commonly used to determine drug purity to control the composition of model binary mixtures in which the second component was admixed at up to 10 mole % of the main component. Using the data determined from the DSC curves (*T_on_*, *T_p_*, and *ΔH_f_*), the content in mole % of the main components in the mixtures analyzed, i.e., 5-dinitrobenzoic acid/benzoic acid, paracetamol/p-aminobenzoic acid, and acetylsalicylic acid/salicylic acid, was calculated based on the van’t Hoff equation. Since the content of the main ingredient calculated from the van’t Hoff equation correlates with its content in the model mixture, the above method can be used to control the composition of binary mixtures with active ingredients. It should be mentioned that APIs must form a simple eutectic system without the possibility of chemical interaction.

The success in determining active ingredients in simple binary mixtures has prompted attempts to estimate the content of APIs in commercially available drug products. Blader Ceipidor et al. [41] developed a simple and reliable method for determining cholic acids (agents used for the treatment of inborn errors of primary bile acid synthesis) in drug products based on the melting heat of analytes determined from DSC curves. Based on the correlation of the melting heat (*y*) with the mass of the sample (*x*), calibration curves were obtained for the five acids analyzed: cholic, chenodeoxycholic, deoxycholic, ursodeoxycholic, and lithocholic. The calibration curves passed through the origin of the coordinate system (*y = a + bx*) and were characterized by high values of correlation coefficients (*R*), from 0.997 to 0.999, and coefficients of variation (*CV*), from 1.6 to 4.5%. This allows the determination of the cholic acids analyzed to range from 0.5 mg for ursodeoxycholic acid to 7.0 mg for cholic, chenodeoxycholic, and deoxycholic acids. The practical utility of the calibration curves was checked by determining the content of chenodeoxycholic and ursodeoxycholic acids in commercial capsules. The results were also compared with those obtained by the enzymatic methods. It was found that the reproducibility of determinations is good, but both methods lead to underestimated results in the case of ursodeoxycholic acid determination. This is likely the result of an unidentified interaction between the components. The studies presented here suggest using the DSC method for the analysis of drug products, as it allows direct determination of cholic acids without separation, while the enzymatic method is suitable for the analysis of biological material, after separation of cholic acids.

Aiming to use DSC also for quantitative analysis of biological material, Marino et al. [42] used a procedure involving thin-layer chromatography (TLC) to separate a mixture of cholic acids, followed by DSC to determine the content of individual cholic acids contained in each spot. Separation of the mixture of cholic, chenodeoxycholic, deoxycholic, and lithocholic acids in the concentration range of 25 to 100 µg of each acid was performed on alumina plates using double elution in the same direction. The gel corresponding to a spot of a particular acid was transferred to the DSC pan and analyzed. The area of the exothermic DSC peak associated with the oxidation of the organic part of the analyte as a function of its mass (*R* = 0.94–0.99) was used to construct the calibration curve. The method has high reproducibility, and the maximum measurement error does not exceed 2%. A procedure involving the use of TLC and DSC was previously described by D’Ascenzo [43] and used for the quantitative analysis of a mixture of amino acids.

Blader Ceipidor et al. [44] compared the DSC method with other procedures used to determine cholic acid in various samples. The data summarized in Table 3 show that the methods studied have satisfactory correlation coefficients (*R*), high precision (*CV*), and accuracy. The enzymatic method has the most favorable parameters, but the chemical method is the most sensitive. In addition, unlike the enzymatic method, the DSC and chemical methods do not require the use of expensive or hazardous reagents. In general, the compared methods for the determination of cholic acid are equally valuable. DSC and chemical methods can be recommended for the analysis of samples of defined composition, with determinations of the heat of oxidation being useful at low analyte contents.

An interesting example of the determination of active ingredient content in tablets was described by Bucci et al. [45]. DSC and TGA curves showed that the sodium salt of diclofenac (an agent used for the treatment of rheumatoid arthritis) is anhydrous and stable during heating, and only undergoes thermal degradation in the temperature range of 265–655 °C with the formation of sodium carbonate. Since its acid form (diclofenac acid) shows a characteristic narrow endothermic peak on the DSC curve (*T_p_* = 182 °C), diclofenac sodium was converted to the free acid form using dilute hydrochloric acid. Based on the calibration curve (heat of conversion from the DSC peak as a function of the mass of precipitated diclofenac acid), diclofenac sodium in Voltaren tablets was quantified with satisfactory precision and accuracy. However, the DSC determination proved more complicated than the UV spectrophotometric determination.

The carried-out studies authorize the conclusion that the use of DSC in the quantitative analysis of drug products depends on the fulfillment of two basic conditions: (a) the presence of the active ingredient in the dosage forms must reflect a characteristic heat effect with an appropriate shape, and (b) the other components of the dosage forms must not affect the area of this peak [46]. This is confirmed by a DSC analysis of three commercial dosage forms of Voltaren, which showed that in the absence of excipients’ influence on the endothermic DSC peak associated with the melting of diclofenac acid (*T_p_* = 182 °C), the linear relationship between peak area and API content in dosage forms makes it possible to determine the active ingredient in soluble tablets, suppositories, and vials.

**Figure 7:**
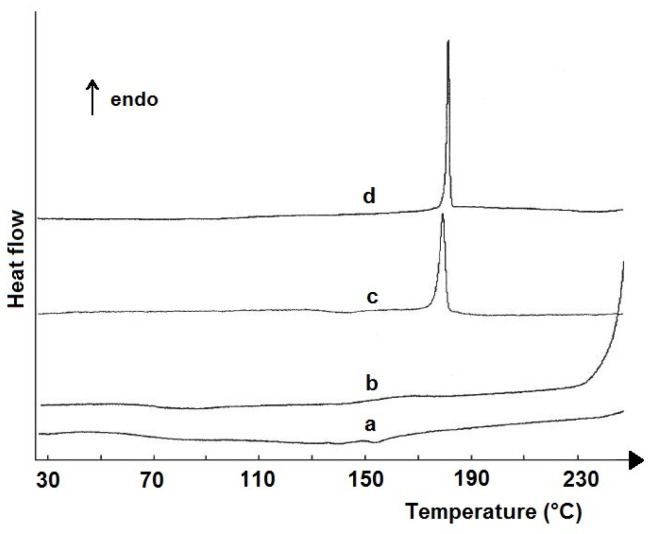
DSC curves of: a—sodium salt of diclofenac, b—nitrilotriacetic acid, c—diclofenac sodium after addition of nitrilotriacetic acid, d—acidic form of diclofenac. Reprinted with permission from Ref. [46], 2025, Springer.

Of the dosage forms mentioned, only soluble tablets contain diclofenac in the free acid form, while suppositories and vials contain diclofenac in the sodium salt form. Therefore, for suppositories and vials, an indirect method must be used, i.e., the sodium salt of diclofenac must be converted to the free acid form (diclofenac acid) using nitrilotriacetic acid (NTA), as illustrated in Figure 7. NTA is a stronger acid than diclofenac, and its excess does not interfere with the assay, as it is thermally inactive up to 225 °C. The method’s recovery, averaging 99.8%, confirms the quantitative conversion of diclofenac sodium to the acid form and indicates that the large excess of NTA, 30 times that of sodium, has no effect on the DSC peak area, except for its slight broadening and shift toward lower temperatures.

Soluble tablets require no pretreatment other than pulverization. Excipients also do not interfere with the assay, as they do not undergo any thermal process up to 250 °C. For suppositories, the suppository base must be extracted with cyclohexane, and NTA must be added to the extraction residue and analyzed by DSC. For vials, it is necessary to evaporate the solution to dryness at ~80 °C, and then DSC analysis is performed after adding NTA. The results of API determinations in the dosage forms analyzed largely coincided with the content declared by the manufacturers. The determined average API contents were, for soluble tablets: 50.82 mg (101.64% of the manufacturer’s declared value, *SD* = 0.89), for suppositories: 100.61 mg (100.61%, *SD* = 0.98), and for vials: 76.81 mg (102.41%, *SD* = 0.71), where *SD* is the standard deviation. The data obtained has high precision and accuracy, and the determination is quite fast, easy to perform, and inexpensive.

A linear relationship between enthalpy of melting and API mass in the range of 2–20 mg was also used by Campanella et al. [47] to determine the paracetamol content of marketed drug products used as analgesics. The presence of excipients ranging from 5 to 15% in the four dosage forms examined had no effect on the endothermic DSC peak due to paracetamol melting. In contrast, the high 65% content of mannitol as excipient and the similar melting temperatures of API and excipient resulted in increased enthalpy of melting values for paracetamol in the drug product, relative to the enthalpy of paracetamol used as a standard. This eliminated the possibility of quantitative analysis of the fifth marketed product. Prior to determining the calibration curve, an endothermic DSC peak deconvolution was performed to separate the peaks associated with slight sublimation preceding paracetamol melting, paracetamol melting, and evaporation of the molten API. The obtained assay results were close to the values declared by the manufacturers. However, it was noted that the lack of certified reference materials makes it impossible to determine the accuracy of the developed method. Using the method of adding a standard (paracetamol) to the analyzed drug products, it was found that the recovery of the DSC method is not less than 96.5%.

A study by Talik et al. [48] showed that the results of paracetamol determinations in commercially available drug products are affected not only by the type of excipients, but also by how they are mixed. Therefore, five calibration curves were determined in the study (the enthalpy of melting of API as a function of its mass) based on DSC curves for paracetamol as a standard and its mixtures with microcrystalline cellulose and corn starch. Paracetamol accounted for 30 to 90% of the mixtures. Two mixtures were prepared as non-micronized and two as micronized samples. The calibration curves obtained were linear in a different range, which included a range from 0.52 to 6.00 mg, while the correlation coefficients were high (*R* = 0.9974–0.9990). On the basis of these curves, the paracetamol content of three marketed products, Paracetamol (Aflofarm, Pabianice, Poland), Codipar (GlaxoSmithKline, London, UK), and Paracetamol (Polfa Lodz, Lodz, Poland), was determined.

The results differed significantly from each other due to the different calibration curves used to calculate them and from the manufacturers’ declared paracetamol content (500 mg). Using the calibration curves for non-micronized mixtures, a lower API content was obtained for the tablets examined than for the calibration curves for micronized mixtures. In addition, the presence of microcrystalline cellulose in the mixture led to more underestimation than corn starch. In contrast, the calibration curve for paracetamol (without excipients) led to intermediate results. The data obtained indicate that the process of manufacturing dosage forms (type of excipients, degree of micronize of the API) affects the results of analytical determinations. This was also confirmed by another study on the effect of micronize on the results of ibuprofen (a non-steroidal anti-inflammatory drug) determinations in six commercial drug products [49].

**Figure 8:**
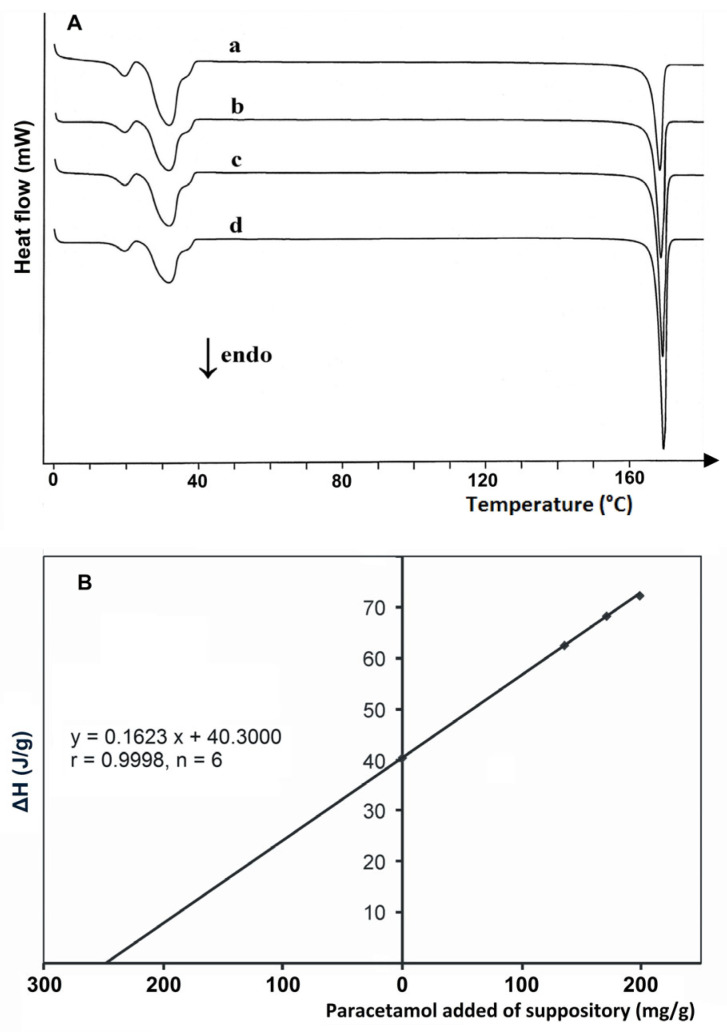
Quantification of paracetamol in Paracetamol 250 mg suppositories (Hasco-Lek). (**A**) DSC curves of suppositories: a—before addition of an adequate quantity of paracetamol as a standard, b—after addition of 100 mg, c—200 mg, and d—300 mg of paracetamol. (**B**) Plot for the multiple standard addition method of paracetamol quantification in suppositories. Reprinted with permission from Ref. [50], 2025, Polish Pharmaceutical Society.

The DSC method was also used to determine paracetamol in rectal dosage forms (suppositories). Leyk et al. [50] analyzed 19 marketed suppositories containing paracetamol in quantities ranging from 50 to 1000 mg. As shown in Figure 8, determinations were made using a multiple standard addition method based on a well-defined endothermic DSC peak due to the melting of paracetamol at *T_on_* = 168.64 °C (heat of melting = 183.99 J/g). The analytical procedure consisted of melting crushed and homogenized suppositories on a water bath at 55 °C, adding appropriate amounts of paracetamol to the melted mass as a standard, and performing DSC analysis after the sample solidified. With the exception of one drug product containing 50 mg of paracetamol, the coefficient of variation (*CV*) for all other determinations ranged from 0.27 to 2.64%, and the relative error ranged from 0.24 to 12.50%. The study showed that DSC can be used as a simple, specific, and reliable method for the determination of paracetamol in suppositories, without the need for suppository base extraction. The results are consistent with the data obtained by the pharmacopoeial method (UV spectrophotometry).

One of the factors that ensures the effectiveness of drugs is the homogeneity of their composition, which consists of the even distribution of the active ingredient throughout the dosage forms. Even distribution of APIs is extremely important for divided powders, suppositories, and globules. Therefore, Noordin and Chung [51] decided to check the uniformity of distribution of paracetamol in suppositories. A calibration curve was prepared by adding appropriate amounts of paracetamol as a standard to the suppository base melted at 40 °C. Although the relationship between the enthalpy of melting and the amount of standard in the suppository base was linear in the range from 0 to 100% paracetamol and passed through the origin of the coordinate system, the range of 4–25% was chosen for quantification (*R* = 0.9962). It was found that the content of paracetamol in the three sections of the suppository, tip, middle, and base, was not statistically significantly different and was consistent with the results of UV spectrophotometry determinations. The authors noted that DSC determinations are only possible if paracetamol does not form a eutectic mixture with suppository base components.

Campanella et al. [52] also analyzed two marketed drug products containing acetylsalicylic acid. The API content determined from the area of the endothermic DSC peak due to its melting was close to the values claimed by the manufacturer. However, it was detected that the peaks, due to the melting and degradation of acetylsalicylic acid, partially overlapped, so peak deconvolution was performed. This process consisted of experimentally determining the DSC baseline using the TGA measurement results as well. The area of DSC peaks obtained by deconvolution yielded a calibration curve in the range of 3.28–17.1 mg, with a higher correlation coefficient (*R* = 0.9980) and a lower limit of detection (*LOD* = 0.16 mg) compared to the data before deconvolution.

In addition, it was found that DSC allows for determining the content of acetylsalicylic acid in the examined dosage forms with greater accuracy, while obtaining results most similar to the values declared by manufacturers, than pharmacopoeial methods, i.e., UV spectrophotometry and the titration method. The values obtained by the titration method were most similar to the DSC results, but it is a very labor-intensive technique, and the data obtained are highly dependent on the skills and experience of the analyst. Spectrophotometric determinations are relatively inexpensive and fast, but the results deviate the most from the values declared by manufacturers and have the highest standard deviation values.

The experimental conditions used for the quantification of active ingredients in marketed drug products by means of DSC are summarized in Table 4. Since the methods described provide results consistent with those obtained by official (pharmacopoeial) methods, they could also be introduced into the pharmacopoeia. It should be noted that API determination using DSC is also used for purposes other than the control of the composition of final dosage forms. Examples include monitoring with DSC of changes over time in the concentration of piracetam in a freshly prepared drug product in connection with testing its shelf life (expiration date) [53] or determining the composition of eutectic mixtures of paracetamol with psycho active ingredients based on the total enthalpy calculated from the area of overlapping DSC peaks due to the melting of eutectic and the major component [54].

### 5.2. TGA Measurements

The first articles on the application of the TGA technique for the quantification of active ingredients in commercial drug products were published by Margomenou-Leonidopoulou et al. and Otto et al., who determined, respectively, the content of novalgin in Buscopan tablets based on dehydration-related mass loss and γ-hexachlorocyclohexane in Delitex powder based on the evaporation of the analyte from dosage forms [13]. Subsequent studies based on the thermal degradation of 117 commercially available drug products have shown that it is possible to directly determine the content of APIs in dosage forms on the basis of dehydration or decarboxylation and on the basis of mass loss associated with the reaction between the components of the effervescent mixture, the formation of an intermediate degradation product and the evaporation or sublimation of the active ingredient [13]. Excipients may not undergo thermal degradation in the temperature range studied. Using the above processes, the content of APIs was determined in more than 50 of the dosage forms evaluated. Similar results were obtained by examining ointments and suppositories. The results of the determinations show high agreement with the manufacturers’ declared values. Relative error values for most determinations are below 5%, which also indicates good precision of TGA and DTG determinations.

Souza et al. [55] used TGA to determine the amount of calcium present in the form of calcium carbonate in tablets for the treatment of osteoporosis. The TGA study showed that thermal decomposition of calcium carbonate is accompanied by two mass losses. The first, a 2.2% mass loss in the temperature range of 291.4–512.7 °C illustrates the presence of impurities in the sample, while the second, a 40.0% mass loss in the temperature range of 630.2–776.2 °C reflects the decarboxylation of calcium carbonate with the release of carbon dioxide and calcium oxide as a residue (see example in Figure 3). Decarboxylation is also confirmed by the endothermic DTA peak in the temperature range of 669.0–791.9 °C (see example in Figure 1). TGA curves of three commercially available drug products containing calcium carbonate at 250 and 600 mg (20.9 to 33.2% calcium) also confirmed the presence of well-shaped mass loss in the temperature range corresponding to decarboxylation. Therefore, the mass loss associated with the release of carbon dioxide was used to calculate the calcium content of the tablets studied. The results are consistent with determinations by inductively coupled plasma-optical emission spectroscopy (ICP-OES). It should be mentioned that excipients do not interfere with the analysis, as they are thermally degraded at lower temperatures.

A number of studies have shown that the use of TGA for quantification of active ingredients is hampered by physical and chemical interactions between dosage forms. Among other things, they cause overlapping thermal events that cannot be distinguished by changing measurement parameters, such as sample mass, heating rate, type, or flow rate of the purge gas. Therefore, Otero et al. [56] attempted to use chemometrics to determine L-ascorbic acid in non-effervescent tablets using TGA in situations where direct determination of the active ingredient is not possible due to interactions between tablet components. To realize the purpose of the study, multiple linear regression (MLR) with temperature selection by the successive projection algorithm (SPA) was used. The use of SPA makes it possible to remove collinearity in data from TGA curves and reduce noise propagation in the MLR model. The resulting multivariate calibration model was used to determine L-ascorbic acid in quantities ranging from 55.0 to 79.5% in ternary mixtures with water (0.5 to 4.5%) and microcrystalline cellulose (19.5 to 42.5%).

The TGA and DTG curves of the analyzed mixtures showed that their thermal degradation profile is similar to that of TGA and DTG of L-ascorbic acid, but it is not possible to accurately determine the mass losses of API and the temperature ranges at which they occur. The situation is also not improved by changing the measurement parameters (sample mass, heating rate, purge gas). Therefore, chemometric calculations were carried out using data from the TGA curves of the mixtures analyzed (the average value of the mass measurements within each 1 °C temperature interval from 32 to 590 °C), with the mixtures divided into three groups containing five, four, and four calibration, validation, and prediction mixtures, respectively. The data obtained showed that the content of L-ascorbic acid in non-effervescent tablets determined using the MLR-SPA procedure is consistent with the expected values, as evidenced by the high value of the correlation coefficient for this relationship (*R* = 0.91) and the low value of the root mean square error of prediction (*RMSEP* = 0.8%). They are also consistent with the results of determinations by iodometric titration (pharmacopoeial method). In addition, they are slightly better than the results obtained using two more multivariate calibration methods, i.e., MLR, with temperature selection using a genetic algorithm (GA), and partial least squares (PLS), with consumption of the entire temperature range from TGA curves. The RMSEP values for MLR-GA and PLS were 1.5% and 1.0%, respectively.

A TGA chemometric data processing procedure was also used by Khanmohammadi et al. [57] for the simultaneous determination of paracetamol and codeine phosphate in tablets. Two multivariate calibration procedures were used: PLS, referred to as a linear modeling technique, and PLS, with the successive projection algorithm (SPA) for temperature selection. The operation of PLS is to build a model describing the relationship between TGA variables and the content of components in the samples under study by creating a new matrix of latent variables. For this purpose, 15 ternary mixtures were prepared containing paracetamol (280–320 mg), codeine phosphate (7–12 mg), and starch (35–50 mg) as an excipient in the range of concentrations in which they occur in marketed tablets. The data from the TGA curves for the mixtures under study were processed using both multivariate calibration methods, i.e., PLS and PLS-SPA, and then, using the calibration models developed, the content of paracetamol and codeine phosphate in 13 commercial tablets was determined. The results are consistent with data from HPLC determinations of the same tablets. It was further found that the calibration model built by PLS-SPA exhibits better prediction capability than the model built by PLS. This is reflected in the RMSEP values, which, for paracetamol and codeine phosphate, were 1.91% and 0.87% (PLS-SPA), and 12.32% and 1.65% (PLS), respectively. The developed procedure also has the advantage of being able to quantify starch.

In general, chemometric processing of TGA data makes it possible to resolve problems arising from overlapping thermal events related to the degradation of drug product ingredients, allowing their simultaneous determination without the need for sample pretreatment, such as extraction or separation. In addition, TGA is a fast, accurate, and reliable method of quantitative analysis, which allows its use in the pharmaceutical industry as a quality control tool. TGA can be categorized as a so-called green chemistry method, as it does not require the use of any organic solvents and is a cost-effective method.

The original method for determining active ingredients in drug products was developed by Gomes et al. [58,59,60]. In brief, the method consisted of determining the vapor pressure profiles of the active ingredient and the drug product containing it, and then determining a calibration curve showing the relation between vapor pressure and the concentration of active ingredient diluted with excipient. The results obtained by the determination of mebendazole (broad-spectrum anti-helminthic agent) [58], ketoconazole (antiandrogen, antifungal, and anti-glucocorticoid drug) [59], and α-lipolic acid (antioxidant and detoxification agent) [60] are consistent with the values obtained by pharmacopoeial methods. However, the realization of the determinations by the described method is labor-intensive, requiring the recording of TGA curves of active ingredient, its mixtures with microcrystalline cellulose and tablets at several heating rates and in two different gaseous atmospheres, and then using the obtained TGA data to determine the reaction order, activation energy, and respective pressure curves of the API and tablet.

The experimental conditions used for the quantification of active ingredients in commercial drug products using TGA are summarized in Table 5. In addition to the determination of API in final dosage forms, examples are also known of the use of TGA for the measurement of API loading in mesoporous silica nanoparticles [61] or for quantification of undesired polymorphic form in the desired one, that is, pantoprazole sodium monohydrate in sesquihydrate [62].

## 6. Analysis of Non-Compliant Drug Products

Knowledge of the thermal profile of active ingredients and excipients (cuts) can also be used to prevent the undesirable situation of non-compliant drug products entering the pharmaceutical market. These products constitute serious health risks due to the absence of the API, its lower or higher content, or the substitution of the API with another substance that differs in pharmacological activity. Regardless of the chromatographic and spectroscopic techniques, methods of thermal analysis can also be used to detect non-compliant drug products.

Thermal methods such as TGA and DSC were used to identify the laboratories that manufacture and distribute fake Merla products, which were confiscated by police [63]. Merla is a complex by-product produced when cocaine (benzoylmethylecgonine) is obtained from the leaves of certain plant species. Because of its cocaine content, it is used as a potent stimulant, and because of this, fake Merla products are appearing on the illegal market. Determined from TGA curves, mass loss at successive temperatures, heat of conversion from endothermic DSC peaks, and cocaine content assessed by gas chromatography-MS (GC-MS) in 30 Merla samples obtained from Instituto de Criminalistica Leonardo Rodrigues (Brazil), were used to search for similarities and dissimilarities between fake Merla products from different manufacturers. A dendrogram obtained using hierarchical cluster analysis (HCA), a chemometric technique, confirmed the fact that several laboratories producing non-compliant products were in operation. The basis for this conclusion was the large differences in the chemical composition of the non-compliant products under study.

DSC and TGA methods have also been used as a fast, reliable, and time-saving approach for direct qualitative and quantitative analysis of fake cocaine products without any sample pretreatment [64]. Cocaine distributed on the illegal market is often mixed with psychoactive or anesthetic agents or with inert substances that increase its mass. It also happens that fake samples distributed as cocaine contain local anesthetics instead of cocaine. Therefore, qualitative and quantitative analysis of fake cocaine products for forensic purposes requires special techniques that provide information on the physical characteristics and quantities of both cocaine and the other components that can be found in fake products, without the influence of sample pretreatment (e.g., dissolution) or experimental conditions (e.g., temperature program in GC) on the data obtained.

DSC curves of cocaine, caffeine, anesthetics (tropocaine, tetracaine, novocaine, lidocaine, and carbocaine), and excipients (mannitol, lactose, and glucose) showed a characteristic thermal profile for each compound with an endothermic DSC peak due to melting. Cocaine melts at ~200 °C, with the highest enthalpy value (*ΔH_f_* ~ 185.5 J/g) among the studied compounds. The TGA curves proved that the superimposed endothermic peaks occurring after melting can be caused by the degradation of cocaine toward benzoic acid and methylecgonine formation. This is followed by the complete decomposition of cocaine without any residue. However, residues were found after the decomposition of commercial samples, probably due to inorganic components used during cocaine extraction or purification. Since the endothermic DSC peaks due to the melting of cocaine and components are well separated, they can be used to qualitatively identify components in fake samples. In addition, these peaks were also used to quantify cocaine and components mixed with cocaine in fake products. A method was developed based on the linear relationship between the enthalpy of melting (*ΔH_f_*) and the analyte content in the mixture, ranging from 10 to 90%. Quantitative analysis is possible because the endothermic DSC peak due to cocaine melting is clearly separated from the component peaks. The developed procedure allows quantification of cocaine and components in non-compliant products with an error of up to ~4%, with an average error of ~2%.

DSC peaks due to the melting of compounds and eutectic mixtures were used by Boumrah et al. [65] to quantify active ingredients of amphetamine-type stimulant drug products available on the illegal street market. DSC curves of three mixtures consisting of amphetamine sulfate, methamphetamine hydrochloride, or 3,4-methylenedioxy-methyl- amphetamine hydrochloride and caffeine showed that only in the case of the mixture of amphetamine sulfate with caffeine, the endothermic peaks were symmetrical, regardless of the content of the components in the mixture. This makes it possible to quantitatively analyze both components over their entire concentration range, based on calibration curves, with the enthalpy of melting (*ΔH_f_*) being a function of analyte concentration.

However, quantitative analysis of the other two mixtures is impossible due to the metastable crystal transformation of caffeine occurring in a temperature range close to the melting points of methamphetamine hydrochloride and 3,4-methylenedioxy-methyl- amphetamine hydrochloride, as well as the formation of eutectic mixtures with caffeine. Using the heating–cooling–reheating DSC program eliminated the crystal transformation of caffeine, and symmetrical endothermic peaks appeared in the DSC curves of both mixtures. The peak at the lower temperature reflected the melting of the simple eutectic mixture, and the one at the higher temperature reflected the melting of the major component. Therefore, the basis for the quantitative analysis was a Tammann plot illustrating the linear dependence of the enthalpy of melting of the eutectic (*ΔH_f_*) with respect to the mass fraction of the API (*w_1_*) in the eutectic mixture. Calibration curves plotted on this basis were used to determine the content of amphetamine-type drugs in the samples under study. The results are consistent with those obtained by the HPLC-DAD (HPLC-diode array detector) method; however, the error of determination by the DSC method is greater than by the HPLC method. It follows that DSC will be more useful in routine determinations where measurement precision is not a priority.

DSC was also used to examine non-compliant diazepam tablets seized by regulators or police in the United Kingdom [66]. The potential use of DSC to definitively identify APIs in an unknown drug product is based on phase and chemical transformations that are characteristic of benzodiazepines, as well as the excipients they contain. DSC curves of crushed and finely ground 16 non-compliant tablets and two commercial tablets showed that both the incompatibility between the API and excipients, and the change in the degree of crystallinity of the API could affect the resolution of the endothermic DSC peak resulting from diazepam melting. For this reason, the presence of diazepam was not clearly identified in some tablets, including one commercial tablet. On the other hand, high levels of diazepam can be displayed as an intense DSC peak due to melting compared to other unknown tablets. In addition, the characteristic DSC effects due to the melting of some excipients, e.g., lactose and stearic acid, or the crystallization of amorphous lactose, can be considered as the manufacturer’s identification mark.

To obtain more information from the DSC data, a chemometric technique, principal component analysis (PCA), was additionally applied. A two-dimensional scatter plot of the results revealed that separate clusters were formed by tablets consistent with their physical properties, diazepam content quantified by HPLC, or lactose content as the main filler. In conclusion, DSC can be considered a method that generates qualitative thermal data on the active ingredient and excipients, and, supported by PCA, can be used as a fast, efficient, and invaluable tool important for forensic identification of unknown tablets.

Sildenafil, known by the brand name Viagra, is used to treat sexual dysfunction and pulmonary hypertension. As a result, non-compliant and dangerous products consisting of this API have appeared on the market with the intention of improving erectile performance in men. Therefore, Maria and Noordin [67] used DSC as a fast, reliable, and inexpensive tool to test whether products seized by police and authorized officers are free of adulteration. They took advantage of the fact that the endothermic DSC peak due to the melting of sildenafil tablets obtained from a licensed manufacturer is very similar to that observed for sildenafil citrate used as a reference standard. However, the thermal profile of the API may undergo slight modifications, e.g., the melting point may be shifted to lower or higher values in the presence of other drug product ingredients. Therefore, to determine the effect of excipients on the melting profile of the API, several mixtures of sildenafil containing between 5 and 95% lactose were examined using DSC. Most of the mixtures exhibited three endothermic effects: The first is due to the dehydration of lactose monohydrate, the second peak is due to the melting of sildenafil, which overlaps with the third peak, which is due to the melting of anhydrous lactose. Studies have shown that the melting point of sildenafil under the influence of increasing amounts of lactose can be shifted to higher values, especially for a mixture containing 5% sildenafil.

With the exception of one sample, endothermic peaks due to the melting of sildenafil as an adulterant were found in the DSC curves of powders prepared from the seized sachets and capsules. However, the melting temperatures were shifted to lower values to varying degrees, while the enthalpies of melting varied over a wide range of values. The addition of API in the range of 30 to 50% confirmed the presence of sildenafil in non-compliant powders. In summary, DSC can be used to detect sildenafil in non-compliant products and to distinguish between sildenafil in its pure form and in a mixture, while HPLC allows identification of the analyte but does not distinguish the form in which it is present. However, DSC does not allow detection of less than 20% sildenafil in a mixture.

DSC curves of 25 non-compliant Viagra and Cialis tablets seized by the Brazilian Federal Police were used by Santos et al. [68] to search for differences between non-compliant and authentic products in order to develop a simple approach to identifying non-compliant drug products. The main difference between the formulations is in the active ingredients; Viagra and Cialis tablets contain sildenafil citrate and tadalafil, respectively. DSC studies of crushed and homogenized authentic tablets showed that Viagra tablets revealed one endothermic and two very small exothermic DSC effects, attributed to sildenafil citrate melting, microcrystalline cellulose degradation, and API degradation, respectively. The DSC peak due to the melting of sildenafil citrate in Viagra tablets is slightly shifted toward lower values, probably due to the tablet’s excipients. Cialis tablets, on the other hand, showed three endothermic DSC effects presumably related to the excipients—magnesium stearate, hydroxypropyl methylcellulose, and lactose monohydrate, respectively. Since Cialis tablets contained about 70% lactose monohydrate, the thermal effects of the excipient probably covered the endothermic peak due to tadalafil melting. Overall, Viagra tablets exhibited a thermal profile similar to that of the active ingredient, while the thermal profile of Cialis tablets differed from that of the active ingredient due to supplementary endothermic peaks attributed to the excipients, especially of lactose monohydrate.

Regarding the non-compliant products, the DSC curves of the non-compliant Viagra tablets were similar to the authentic ones, except for one sample with an additional exothermic effect. This peak could be attributed to a different excipient than those used in the manufacture of the licensed tablets. On the other hand, some of the non-compliant Cialis tablets showed a similar thermal profile to both authentic and non-compliant Viagra tablets. The additional endothermic peak related to sildenafil melting suggests that some non-compliant Cialis tablets contained sildenafil citrate as the API instead of tadalafil. Other non-compliant Cialis tablets revealed additional DSC peaks that may be related to excipients used in the manufacture of the non-compliant tablets. In addition, using HCA, authentic and non-compliant tablets were grouped into four clusters based on the DSC data. The criteria for including samples in these clusters were as follows: high sildenafil content in non-compliant tablets, the same API and origin of non-compliant tablets, the same sildenafil content as in authentic Viagra tablets, and, presumably, the same content of excipients and API. Thus, the study proved that the HCA-supported DSC provided useful data to distinguish between non-compliant and authentic drug products in a simple and inexpensive way.

The conditions under which studies were performed on the application of thermal methods in the analysis of non-compliant drugs are shown in Table 6. They confirm the crucial importance of endothermic DSC peaks due to melting compounds (or their eutectics) in all aspects of analytical determinations. In contrast, TGA curves can only be useful when their interpretation is supported by chemometric techniques. The great importance of chemometrics in the interpretation of TGA data for quantitative analysis is indicated, among others, by studies in the area of food chemistry [69]. Using PLS to interpret DTG data, adulterants in coffee were quantified.

## 7. General Remarks on Thermal Methods

A review of the literature shows that, out of a large group of thermal analysis methods, only two methods, traditional DSC and TGA, were used for the quantitative analysis of commercially available drug products. Quantitative DSC measurements involved determining the linear relationship between the melting heat of the active ingredient and its content in the drug product. In TGA measurements, on the other hand, the amount of mass loss occurring on the TGA curves within a specific temperature range was compared of the drug product and active ingredient. Quantitative determinations are only possible if the measurements were performed under the same conditions and using the same type of instrument. Furthermore, excipients should not be thermally active in the examined temperature range and must not influence the melting or thermal degradation process of the API being determined.

Despite the many advantages of the analytical procedures described in Section 5, their fundamental drawback is the lack of proper validation. According to the ICH guidelines contained in the document Q2 (R1) [70], the validation of analytical methods used in pharmacy is carried out by determining the most important parameters characterizing a given method, i.e., selectivity, specificity, precision (repeatability, intermediate precision, reproducibility), accuracy (recovery), linearity, measurement range, limit of detection (LOD), limit of quantitation (LOQ), sensitivity, and robustness. None of the cited articles validated the developed method in accordance with ICH requirements. The concern of the highest state authorities for public health means that the methods used to study active pharmaceutical ingredients and drug products and for their quantitative analysis must meet the highest standards. Therefore, the lack of validation prevents the use of procedures involving DSC and TGA as official methods accepted by pharmaceutical law.

Table 7 presents a comparison of the most commonly used chromatographic and spectroscopic methods in the quantitative analysis of drug products with the proposed thermal procedures. Analysis of these data indicates that thermal methods could be a good complement to HPLC and UV-Vis methods, but only after their validation and acceptance by official pharmaceutical authorities.

A review of the scientific literature also shows that, to date, technologically advanced DSC methods such as modulated temperature DSC (MTDSC), high sensitivity DSC (HSDSC), step scan DSC (SSDSC), fast-scan DSC (Fast DSC), high-performance DSC (HyperDSC), and high-pressure DSC (HPDSC) have not yet been used in the quantitative analysis of commercial drug products. The benefits of using these methods are described in the latest book on DSC [19], and their application in pharmacy is confirmed by numerous publications. For example, MTDSC has been used for qualitative and quantitative analysis of polymorphic forms [71], to study the properties of amorphous and crystalline forms [72], co-crystals and co-amorphous complexes [73], and amorphous solid dispersions of APIs [74,75]. Fast DSC has been used for the identification of polymorphic impurities, while HyperDSC and SSDSC have been used for the quantitative determination of amorphous phases of APIs [71]. There is also a lack of data in the scientific literature on the use of coupled (TG-FTIR, TG-MS, and TG-GC) and simultaneous (DSC-photovisual, DSC-FTIR, and DSC-XRD) methods in the quantitative analysis of commercial drug products [17]. The only published example indicates the possibility of using a coupled technique, TG-APPI-MS, for quality control of finished drug products and for detecting drug products from illegal manufacturers [26]. There is, therefore, hope that new analytical procedures for the quantitative determination of APIs in commercially available drug products using technologically advanced thermal analysis equipment may be published.

## 8. Conclusions

Based on the review of the literature, it has been proven that thermal analysis methods DSC, DTA, and TGA can be useful in distinguishing drug products from different manufacturers, which guarantees their usefulness not only in quality control of finished drug products, but also for detecting drug products from illegal manufacturers. They are also useful as tools for confirming the presence of APIs in dosage forms under investigation. Identification of active ingredients is done by comparing the shape of DSC or DTA peaks, as well as mass losses and temperature ranges, with the same parameters on the standard substance curves. The most favorable results are obtained from narrow and high endothermic peaks due to the melting of the API. Taking advantage of the fact that the heat of transformation reflected by the endothermic peak is proportional to the API content of the drug product, the possibility of using DSC in the quantification of APIs in marketed drug products and to detect non-compliant drug products has also been pointed out. Numerous examples also show the usefulness of TGA in quantitative analysis, and the use of chemometric techniques for interpreting TGA data allows for the elimination of the adverse effect of excipients on quantification results.

In general, thermal methods are a good complement to chromatographic and spectroscopic methods. The advantages of using thermal methods in the analysis of the composition of drug products are the lack of the need for special preparation of samples for testing (extraction, separation), low sample mass, and short time for performing analyses, as well as high sensitivity of determinations. The simple way of performing thermal measurements makes it possible to obtain data on the phase transformations occurring in the samples under study and on their thermal degradation, along with the possibility of determining the kinetic parameters and mechanism of this process. An important advantage of thermal methods is also that, as opposed to chromatographic and spectroscopic methods, they do not require the samples to be run into solution. Thus, they make it possible to obtain data on the forms in which APIs are present in the dosage formulations, i.e., amorphous or crystalline form, polymorphic modification, anhydrous form, hydrate, or solvate. In addition to the content of active ingredients, these are key data determining the quality, safety, and efficacy of drugs used by patients.

## Figures and Tables

**Table 1:** Thermal methods of analysis for distinguishing commercial drug products originated from different manufactures.

Commercial Drug Products (Active Ingredients)	Thermal Analysis Devices	Measurement Conditions	Refs.
Tablets, capsules, powders (analgesics, antacids, vitamins)	Model 900 DTA (with DSC cell), Model 950 TGA (Du Pont)	DTA (DSC): 5–10 mg, 25–450 °C, 10 °C/min, nitrogen; TGA: 5–20 mg, 25–450 °C, 10 °C/min, nitrogen	[23,24,25]
Tablets (acetylsalicylic acid)	STA 7200 TG/DTG/DTA (Hitachi)	4–6 mg, 45–550 °C, 10 °C/min, nitrogen 200 mL/min	[26]
Tablets (theophylline, aminophylline)	TG 209 F3 Tarsus (Netzsch)	10 mg, 35–600 °C, 10 °C/min, nitrogen 40 mL/min	[27]
Tablets, coated tablets (naproxen, naproxen sodium)	TG 209 F3 Tarsus (Netzsch)	10 mg, 35–600 °C, 10 °C/min, nitrogen 40 mL/min	[28]

**Table 2:** The application of thermal methods of analysis for qualitative identification of active ingredients in commercial drug products.

Commercial Drug Products (Active Ingredients)	Thermal Analysis Devices	Measurement Conditions	Refs.
Tablets, granulates, capsules, powders, dragees (neuroleptics, expectorants, chemotherapeutics, vitamins, inorganic active ingredients)	Derivatograph OD-130 (MOM, Budapest)	100 mg, 20–600 (1000) °C, 5 °C/min, furnace atmosphere	[13]
Ointments, suppositories (various active ingredients)	Derivatograph OD-130 (MOM, Budapest)	200 mg, 20–600 (800) °C, 5 °C/min, furnace atmosphere	[13]
Tablets (atenolol)	DSC-60, TGA-50, TA-501 Thermal Analyzer (Shimadzu)	DSC: 0.8–1.2 mg, 20–335 °C, 10 °C/min, nitrogen 20 mL/min; TGA: 8–12 mg, 20–600 °C, 10 °C/min, nitrogen 20 mL/min	[30]
Tablets, pellets, capsules, suppositories, dosage forms in development (various active ingredients)	DSC-2, DSC-7, TGA-7 (Perkin-Elmer)	20 °C/min, nitrogen	[31]
Tablets (coated and uncoated), pellets, granulates, capsules, dragees (methylxanthines)	DSC 822e (Mettler Toledo)	4 mg, 25–300 °C, 10 °C/min, nitrogen 70 mL/min	[32]
Tablets (coated and uncoated), effervescent tablets, capsules, dietary supplements (magnesium salts)	DSC 822e (Mettler Toledo)	4 mg, 25–300 °C, 10 °C/min, nitrogen 70 mL/min	[35]
Tablets, effervescent tablets, capsules, sachets (paracetamol)	“Jade” DSC calorimeter (Perkin-Elmer)	3 mg, 30–300 °C, 5 °C/min, nitrogen 70 mL/min	[36]

**Table 3:** Comparison of the methods used for cholic acid determination.

Methods	Measurement Conditions	R	CV, %	Analytical Range
DSC, melting	Heat of melting from endothermic DSC peak in the range of 140–210 °C, dynamic nitrogen atmosphere	0.996	4.5	1.00–6.70 mg
DSC, oxidation	Heat of oxidation from exothermic DSC peak in the range of 220–450 °C, dynamic oxygen atmosphere	0.996	4.0	2.50–10.00 mmole/L
Chemical	Reaction with freshly distilled furfural at 65 °C, absorption measure of final solution at 340 nm	0.998	4.4	0.120–1.250 mmole/L
Enzymatic	Reaction catalyzed by 3α-hydroxysteroid dehydrogenase, absorption measure of final solution at 750–760 nm	0.999	2.4	1.00–5.00 mmole/L

**Table 4:** DSC quantification of active pharmaceutical ingredients in commercially available drug products.

Commercial Dosage Forms (Active Ingredients)	Thermal Analysis Devices	Measurement Conditions	Refs.
Capsules (chenodeoxycholic acid, ursodeoxycholic acid)	DSC-2 (Perkin-Elmer)	4–5 mg, 2.5 °C/min, nitrogen 100 mL/min	[41]
Tablets, soluble tablets, suppositories, vials (diclofenac, diclofenac sodium)	DSC-7 series 1020 and TGS-2 (Perkin-Elmer)	DSC: 0.3–0.5 mg, 20–360 °C, 10 °C/min, nitrogen 50 mL/min; TGA: 1–2 mg, 20–840 °C, 10 °C/min, nitrogen 50 mL/min	[45,46]
No dosage form specified (paracetamol)	TG/DSC 625 (Stanton-Redcroft)	4–6 mg, 20–400 °C, 2.5, 5, 10, 20 °C/min, argon	[47]
Tablets (paracetamol)	EXSTAR DSC 7020 (SII Nano Technology Inc.)	4.9 mg, 30–190 °C, 10 °C/min, nitrogen 50 mL/min	[48]
Tablets (ibuprofen)	EXSTAR DSC 7020 (SII Nano Technology Inc.)	5.0 mg, from 30 °C, 10 °C/min, nitrogen 50 mL/min	[49]
Suppositories (paracetamol)	DSC 822e (Mettler Toledo)	5.0 mg, 10–280 °C, 5 °C/min, nitrogen 80 mL/min	[50]
Suppositories (paracetamol)	DSC-6 (Perkin-Elmer)	2.5–4.0 mg, 0–180 °C, 5 °C/min, nitrogen 20 mL/min	[51]
No dosage form specified (acetylsalicylic acid)	TG/DSC 625 (Stanton-Redcroft)	7 mg, 20–600 °C, 10 °C/min, air 50 mL/min	[52]

**Table 5:** TGA quantification of active pharmaceutical ingredients in commercially available drug products.

Commercial Dosage Forms (Active Ingredients)	Thermal Analysis Devices	Measurement Conditions	Refs.
Tablets (calcium carbonate)	DTG 60 (Shimadzu)	7 mg, 20–900 °C, 10 °C/min, air 50 mL/min	[55]
Non-effervescent tablets (L-ascorbic acid)	TGA-2950 (TA-Instruments)	10 mg, 30–600 °C, 10 °C/min, oxygen 100 mL/min	[56]
Tablets (paracetamol, codeine phosphate)	Pyris Diamond TG/DTG (Perkin-Elmer)	25–900 °C, 10 °C/min, nitrogen 100 mL/min	[57]
Tablets (mebendazole, ketoconazole)	DSC-50 and TGA-50H (Shimadzu)	10, 20, 40, 60, 80 °C/min; DSC: 25–500 °C, nitrogen 50 mL/min; TGA: 8 mg, 25–900 °C, synthetic air 20 mL/min, nitrogen 50 mL/min	[58,59]
Capsules (α-lipolic acid)	TG/DTA model Q600 (TA-Instruments)	5.0 mg, 25–900 °C, 10, 20, 40, 60, 80 °C/min, nitrogen 50 mL/min	[60]

**Table 6:** The application of thermal methods of analysis in the study of non-compliant products.

Non-Compliant Products (Active Components)	Purpose of Research	Thermal Analysis Devices	Measurement Conditions	Ref.
Merla (cocaine)	Difference in chemical composition	DSC 822e and TGA/SDTA 851e (Mettler Toledo)	DSC: 5 mg, 25–600 °C, 10 °C/min, nitrogen 50 mL/min; TGA: 7 mg, 25–1400 °C, 50 °C/min, nitrogen 50 mL/min	[63]
Powders (cocaine)	Identification and quantification	DSC-2B and TG-S2 (Perkin-Elmer)	between 2.5 and 10 °C/min, nitrogen or air 50–100 mL/min	[64]
Powders (amphetamine-type drugs)	Quantification	DSC 8000 (Perkin-Elmer)	2–5 mg, 10 °C/min, nitrogen 20 mL/min	[65]
Tablets (benzodiazepines, excipients)	Identification	DSC Q 100 (TA Instruments)	5–10 mg, 20–250 °C, 10 °C/min, nitrogen 50 mL/min	[66]
Tablets, capsules, sachets, powders (sildenafil, excipients)	Detection	DSC 6000 (Perkin-Elmer)	0.5–1.0 mg, 100–200 °C, 10 °C/min, nitrogen 20 mL/min	[67]
Tablets of Viagra and Cialis (sildenafil, tadalafil, excipients)	Discrimination between different tablets	DSC-60 (Shimadzu)	1–2 mg, 30–330 °C, 10 °C/min, nitrogen 50 mL/min	[68]

**Table 7:** Comparison of thermal, chromatographic, and spectroscopic methods from the perspective of quantitative analysis of commercially available drug products.

Drug Products (e.g., Tablets, Capsules, Suppositories)	Thermal Methods (DSC, TGA)	Chromatographic Methods (HPLC)	Spectroscopic Methods (UV-Vis)
Time of analysis ^a^	minutes	hours	hours
Labor-intensity of analysis	negligible ^b^	high ^c^	high ^c^
Cost of analysis	low ^d^	high ^e^	high ^e^
Transferring the sample into solution	not needed	necessary	necessary
Separation of analyte from matrix	not needed	necessary	necessary
Use of solvents and reagents	not needed	necessary	necessary
Problems with stability of analyte	no	yes	yes
Sample analysis in solid state	yes	no	no
Solid state properties of analyte ^f^	yes	no	no
Green chemistry method	yes	no	no

a—time from the sample acquisition to the calculation of the analysis result; b—grinding the sample in a mortar; c—transferring the sample to the solution, using various techniques to separate and purify the analyte; d—cost of the apparatus; e—costs of the apparatus, laboratory equipment, solvents, and reagents; f—make possible to obtain data on the forms in which analyte is present in the drug form, i.e., amorphous or crystalline form, polymorphic modification, anhydrous form, hydrate or solvate, acidic or salt form.

## Data Availability

Not applicable.

## Abbreviations

The following abbreviations are used in this manuscript:

API	Active pharmaceutical ingredients
DSC	Differential scanning calorimetry
DTA	Differential thermal analysis
TGA	Thermogravimetric analysis
